# Predictive Value of tTG-IgA ≥ 10×ULN for Villous Atrophy in a Romanian Adult Cohort: The Modifying Role of Smoking

**DOI:** 10.3390/diagnostics16060838

**Published:** 2026-03-12

**Authors:** Roxana Nemteanu, Irina Girleanu, Alexandru-Ionut Coseru, Irina Ciortescu, Mihaela Dranga, Otilia Nedelciuc, Vasile-Andrei Olteanu, Anca Trifan, Florentina Severin, Andreea Clim, Mihai Danciu, Laura Otilia Boca, Alina Plesa

**Affiliations:** 1 Department Medicale I, “Grigore T. Popa” University of Medicine and Pharmacy, 700454 Iași, Romania; maxim_roxxana@yahoo.com (R.N.); gilda_iri25@yahoo.com (I.G.); ionutz_ionutz_barlad@yahoo.com (A.-I.C.); mihaela_dra@yahoo.com (M.D.); otilianedelciuc@yahoo.com (O.N.); olteanuandrei@yahoo.com (V.-A.O.); anca.trifan@yahoo.com (A.T.); florentina.s.severin@umfiasi.ro (F.S.); andreea.clim13@yahoo.com (A.C.); mihaida2@yahoo.com (M.D.); laura.boca@umfiasi.ro (L.O.B.); alinaplesaro@yahoo.com (A.P.); 2 Institute of Gastroenterology and Hepatology, “Saint Spiridon” University Hospital, 700111 Iași, Romania; 3 Ear, Nose, and Throat Clinic Department, “Saint Spiridon” University Hospital, 700111 Iași, Romania; 4 Pathology Department, “Saint Spiridon” University Hospital, 700111 Iași, Romania; 5 Saint Mary Emergency Children Hospital, 700309 Iași, Romania

**Keywords:** celiac disease, tTG-IgA, duodenal biopsy, biopsy-free diagnosis, smoking, villous atrophy

## Abstract

**Introduction:** Celiac disease (CD) is a prevalent autoimmune enteropathy that remains significantly underdiagnosed due to its multifaceted diagnostic pathway and diverse clinical presentations. While duodenal biopsy has historically served as the diagnostic gold standard, its clinical primacy has been challenged by the burden of invasive endoscopy and potential histological misinterpretation. **Material and Methods:** We conducted a prospective diagnostic accuracy study involving consecutive adult patients with suspected CD to evaluate the performance of serological markers against histological findings. **Results:** The study included 139 patients, with a female predominance of 105 (75.5%). Histological evaluation revealed Marsh 3a–c in 100 patients (71.9%), whereas Marsh 1–2 was observed in 39 patients (28.1%). Sixty-one patients (43.9%) presented with high-titer ≥10×ULN tTG-IgA levels, while 78 patients (56.1%) fell below this threshold. To determine the independent predictors of Marsh 3a–c, we performed a logistic regression analysis. In the univariate analysis, both tTG-IgA (OR: 1.880; 95% CI: 1.458–2.426; *p* < 0.001) and non-smoker status (OR: 1.865; 95% CI: 1.283–2.709; *p* = 0.002) were significantly associated with VA. After adjusting for confounding variables in the multivariate model, both factors remained highly significant (*p* < 0.001 and *p* = 0.014, respectively). The diagnostic performance of the ≥10×ULN tTG-IgA threshold for detecting VA was confirmed by AUROC of 0.737, CI 0.646–0.827, *p* < 0.001, with a sensitivity of 55.0% and a specificity of 84.6%, a PPV of 90.2% and a NPVof 42.3% (33/78), underscoring that while the ≥10×ULN tTG-IgA threshold is highly specific for atrophy, lower titers do not reliably exclude it in adults. **Conclusions:** In conclusion, our study demonstrates that the ≥10×ULN tTG-IgA threshold provides a reliable diagnostic surrogate for VA in adult CD. While high cut-off values minimize false positives, a diagnostic gap remains for patients with lower antibody levels or those influenced by modifiers such as smoking. The low sensitivity of the high threshold reinforces the continued necessity of duodenal biopsy for symptomatic patients with lower-range antibody elevations to avoid a significant diagnostic gap.

## 1. Introduction

Celiac disease (CD) is a prevalent autoimmune enteropathy occurring in genetically predisposed individuals [1,2]. In susceptible patients, gluten ingestion triggers villous atrophy (VA), micronutrient deficiencies, and a diverse spectrum of clinical phenotypes [2]. The multifaceted, multi-step diagnostic pathway, coupled with the necessity for high clinical suspicion, remains a core determinant for the widespread underdiagnosis of CD [3]. Although duodenal biopsy was historically the “gold standard” for CD diagnosis, its clinical primacy has declined in recent years [4]. This shift gained momentum in 2012 when the European Society for Paediatric Gastroenterology, Hepatology and Nutrition (ESPGHAN) introduced a biopsy-free diagnostic protocol for symptomatic children meeting rigorous criteria. These include an elevation of immunoglobulin A (IgA) anti-tissue transglutaminase (tTG) ≥10 times the upper limit of normal (ULN) and a positive anti-endomysium antibody (EMA) test from a separate blood draw [5]. This pivot was supported by emerging data demonstrating that the specificity of tTG-IgA for duodenal VA correlates positively with the magnitude of antibody elevation in pediatric populations.

Since its inception, the biopsy-free approach has gained global traction [6]. Clinicians have highlighted several concerns regarding traditional diagnostics, including the burden of endoscopy—which is invasive, costly, and carries inherent risks—as well as the limitations of histology [7]. Factors such as improper biopsy sampling, orientation, and staining are time-consuming and operator-dependent, and often lead to histological misinterpretation. Furthermore, the histological classification of CD has seen no significant refinements in the past decade to improve its predictive accuracy [8].

To date, substantial evidence supports extending the no-biopsy approach to the adult population when utilizing the ≥10×ULN tTG-IgA threshold [6,9]. Consequently, the American College of Gastroenterology (ACG) updated its 2023 guidelines to include the biopsy-free approach as a conditional recommendation [4]. However, professional reluctance persists regarding the application of these serological criteria in adults. This hesitancy stems from the risk of false negatives in low-prevalence populations (e.g., asymptomatic patients or those with autoimmune comorbidities) or individuals on a low-gluten diet who may not reach the required serological threshold [9]. While high cut-off values minimize false positives, a diagnostic gap remains for patients with lower antibody levels, who would still derive significant benefit from a confirmatory duodenal biopsy [10].

A primary candidate for this discordance is smoking status. Tobacco use has long been recognized as a unique modifier in CD; epidemiological data suggest an inverse relationship between smoking and the risk of developing CD, a phenomenon often termed the “smoking paradox” [11]. Mechanistically, it is hypothesized that nicotine or other tobacco components may exert an immunosuppressive effect on the intestinal mucosa or reduce epithelial permeability, potentially masking the progression of VA despite high levels of systemic autoimmunity [12]. Beyond smoking, several other factors contribute to the serological-histological gap in adults, such as patchy atrophy (as opposed to a more uniform mucosal damage seen in children), fluctuations in gluten intake, comorbidities, medication use, and aging [13]. Given these variables, there is a critical need to validate the ≥10×ULN tTG-IgA threshold in specific adult cohorts. Understanding how factors such as smoking status influence diagnostic accuracy is essential for moving toward a more personalized and reliable non-invasive diagnostic framework.

The study was designed on the hypothesis that a ≥10×ULN tTG-IgA threshold maintains high specificity for VA in adult cohorts. Furthermore, we posited that smoking status acts as a significant modifier, correlating with an increased prevalence of discordance between serological magnitude and histological severity.

## 2. Materials and Methods

### 2.1. Study Design

This study is designed as a prospective diagnostic accuracy study. To ensure transparent reporting, we followed the STARD guidelines. The study was conducted at a tertiary referral center in Romania between January 2014 and December 2025. The participants were enrolled consecutively upon clinical suspicion of CD. All patients underwent serological testing (serum tTG-IgA levels, expressed as multiples of the upper limit of normal) followed by endoscopy and histopathological examination of at least 4–6 samples graded according to the Marsh–Oberhüber classification. The index test (tTG-IgA) and the reference standard (duodenal biopsy) were performed as part of a single diagnostic episode, with a maximum time interval of 14 days between blood sampling and endoscopy to ensure that patients did not initiate a gluten-free diet before the biopsy was completed. The ≥10×ULN tTG-IgA threshold was defined a priori, in accordance with international guideline recommendations, and was used as the primary predictor in all diagnostic accuracy and regression. Patients were included if they were 18 years or older and provided written informed consent at the time of screening. We rigorously applied exclusion criteria at the point of enrollment, including concurrent gastrointestinal infections, malignancy, tuberculosis, Crohn’s disease, or incomplete medical records. Following inclusion, clinical and laboratory parameters were systematically collected and documented following a standardized sequential protocol initiated by serological screening and followed by confirmatory small bowel biopsy. Smoking status was assessed at the time of enrollment and categorized into two primary groups for statistical power: “Never smokers” (defined as individuals who had smoked fewer than 100 cigarettes in their lifetime) and “Smokers”. The smoker group comprised current active smokers and former smokers who had ceased tobacco use within the 12 months prior to enrollment.

### 2.2. Diagnostic Criteria

The diagnosis of CD in symptomatic individuals followed a sequential protocol, initiated by serological screening and confirmed via small bowel biopsy. The age at diagnosis was defined as the date of the initial pretreatment intestinal biopsy. All clinical and laboratory data were assessed at the time of diagnosis. The study protocol received approval from the local institutional ethics committee and was conducted in compliance with the Declaration of Helsinki.

### 2.3. Laboratory and Serological Assessment

Serological markers were determined using a commercial enzyme-linked immunosorbent assay (ELISA) kit, Euroimmun, Lubeck, Germany, with a positive threshold defined as >15 IU/mL. To ensure cross-assay comparability, results were expressed as multiples of the assay-specific ULN, calculated as the ratio of the patient’s absolute concentration to the laboratory’s standard upper limit

Total serum IgA levels were measured to exclude IgA deficiency (defined as a concentration below 0.07 g/L). In cases where tTG-IgA titers were ≥10×ULN but histology showed only mild enteropathy (Marsh 1–2), clinical and serological follow-up was initiated. These patients were managed as potential CD cases according to international guidelines. For the purpose of this diagnostic accuracy analysis, they were categorized based on their initial biopsy result as non-atrophy (Marsh 1–2). Indeterminate serological results were repeated for confirmation before inclusion.

### 2.4. Histological Evaluation

Duodenal samples were obtained via esophagogastroduodenoscopy (EGD), with a mean of 3.4 ± 0.8 biopsies per patient. To ensure diagnostic accuracy, samples were collected from multiple sites, including at least one from the duodenal bulb and a minimum of two from the distal duodenum. Tissue samples (4 µm-thick, paraffin-embedded) were processed with hematoxylin and eosin, periodic acid-Schiff, and immunohistochemical staining for CD3. Histological changes were assessed by two independent experienced gastrointestinal pathologists and graded according to the Marsh–Oberhüber classification, utilizing an intraepithelial lymphocyte (IEL) threshold of >25 IELs/100 enterocytes. Marsh 1–2 lesions were defined by increased IELs, without or with crypt hyperplasia, respectively, and normal villous architecture, while Marsh 3a–c denoted progressive degrees of VA. In cases of architectural heterogeneity (patchy lesions), the highest Marsh grade observed was recorded as the definitive score. In instances of architectural heterogeneity or grading discrepancy, a consensus adjudication was reached between the two observers through simultaneous review using a multi-head microscope to determine the definitive score.

### 2.5. Statistical Analysis

Statistical analysis was conducted utilizing SPSS version 26.0 (SPSS Chicago Inc., Chicago, IL, USA). The required sample size was determined based on the expected specificity of the ≥10×ULN tTG-IgA threshold for detecting VA. Assuming a target specificity of 85% based on previous adult literature, a 95% confidence level, and a 10% margin of error, a minimum of 49 patients with histological VA was required. Our final cohort included 100 patients with confirmed VA (Marsh 3a–c) and a total of 139 participants, providing sufficient statistical power to validate the diagnostic accuracy and the independent predictors identified in the multivariate logistic regression model. Continuous variables were presented as mean ± standard deviation (SD) or medians with interquartile ranges (IQR), contingent upon the distribution’s normality, which was evaluated via the Kolmogorov–Smirnov test. Categorical variables were displayed as absolute frequencies and percentages. TTG-IgA was modeled using a prespecified binary cut-point (≥10×ULN vs. <10×ULN) in the primary multivariable logistic regression analysis. This approach was selected a priori to directly evaluate the clinical utility of the guideline-recommended threshold for biopsy-free diagnosis, consistent with ESPGHAN and ACG recommendations [2,4]. Although tTG-IgA values were available as continuous multiples of ULN, the primary objective of the regression model was to assess the independent predictive value of the ≥10×ULN threshold in relation to villous atrophy (Marsh 3a–c). Continuous modeling of tTG-IgA was explored in univariate analysis but was not retained in the final multivariable model to avoid collinearity with the binary threshold variable and to preserve clinical interpretability. The dependent variable in all logistic regression models was villous atrophy, defined as Marsh 3a–c (yes/no), with Marsh 1–2 serving as the reference category. The following variables were evaluated as potential predictors based on biological plausibility and clinical relevance: tTG-IgA ≥10×ULN (binary, age, sex, smoking status, hemoglobin, total serum IgA). All variables were first assessed in univariate logistic regression analyses. Variables with *p* < 0.20 in univariate analysis were considered candidates for the multivariable model. Missing data were minimal (<2% across variables). Given the very low proportion and absence of systematic missingness, we performed a complete case analysis. No imputation procedures were required.

Receiver Operating Characteristic (ROC) curve analysis was conducted to assess the overall discriminatory capacity of tTG-IgA (continuous) for predicting VA, expressed as Area Under the Curve (AUC). The prespecified 10×ULN threshold was subsequently plotted on the ROC curve to illustrate its diagnostic performance; no data-driven threshold optimization was performed. Group comparisons were conducted using the Student’s *t*-test or Mann-Whitney U test for continuous variables, and the Chi-square test or Fisher’s exact test for categorical data.

## 3. Results

**Figure 1:**
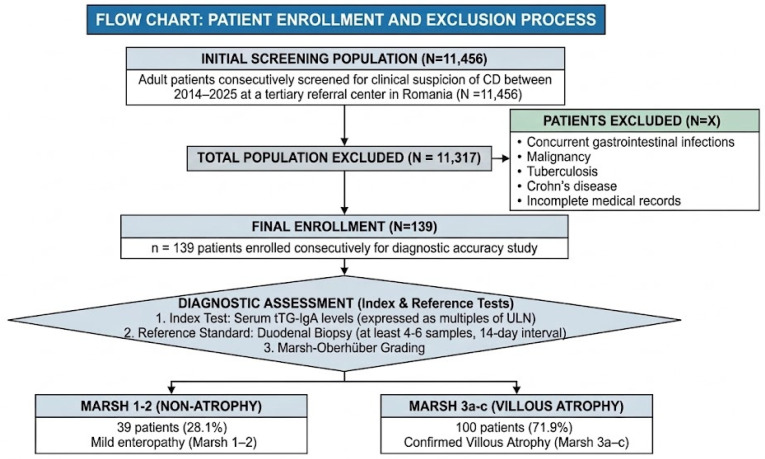
Flow-chart of patient enrolment process.

A total of 139 patients fulfilled the inclusion criteria and were subsequently included in the investigation (Figure 1). The cohort exhibited a female predominance (*n* = 105; 75.5%) with a mean age of 41.6 + 12.9 years (range: 18–73 years).

The smoker status was assessed, and 82 (59%) were smokers, while 57 (41%) had never smoked. The majority of patients presented with gastrointestinal symptoms such as abdominal pain in 102 cases (74.8%), recurrent diarrhea in 88 cases (63.3%), and abdominal distension in 74 cases (53.2%). Iron deficiency anemia was detected in 87 cases (62.6%), while dermatitis herpetiformis was documented in 7 cases (5%). Thyroiditis was the most prevalent autoimmune condition, with a majority of 15 cases (10.7%). Psychiatric disorders (anxiety, depression, bipolar disease, and schizophrenia) were also diagnosed in 18 cases (12.9%). No patient was IgA-deficient in the cohort. All patient characteristics are listed in Table 1.

Histological evaluation revealed advanced mucosal damage (Marsh 3a–c) in 100 patients (71.9%), whereas mild enteropathy (Marsh 1–2) was observed in 39 patients (28.1%) (Table 2). Among those with VA, the distribution was as follows: Marsh 3a (*n* = 36; 25.9%), Marsh 3b (*n* = 24; 17.3%), and Marsh 3c (*n* = 45; 32.4%). Regarding serological titers, 61 patients (43.9%) presented with high-titer ≥10×ULN tTG-IgA levels, while 78 patients (56.1%) fell below this threshold. The transition from mild enteropathy to VA was accompanied by significant biochemical shifts. Patients expressing VA were more likely to develop anemia (11.5 ± 2.11 vs. 11.7 ± 2.49, *p* = 0.002), vitamin D deficiency (36.66 ± 41.01 vs. 37.59 ± 79.17, *p* = 0.030), calcium deficiency (9.08 ± 0.68 vs. 8.75 ± 0.68, *p* = 0.013), and hypoalbuminemia (4.02 ± 0.56 vs. 3.71 ± 0.67, *p* = 0.013). No differences were detected when comparing mild enteropathy vs. VA with regard to intestinal and extraintestinal manifestations. These findings suggest that while serology indicates the autoimmune drive, these malabsorptive markers remain essential indicators of the functional impact of the intestinal lesion.

To determine the independent predictors of advanced mucosal damage (Marsh 3a–c), we performed a logistic regression analysis. In the univariate analysis, both tTG-IgA ≥10×ULN threshold (OR: 1.880; 95% CI: 1.458–2.426; *p* < 0.001) and never-smoked status (OR: 1.865; 95% CI: 1.283–2.709; *p* = 0.002) were significantly associated with VA. In the multivariable analysis (Table 3), two factors emerged as independent predictors of VA. After adjusting for age and gender, tTG-IgA titers ≥10×ULN were associated with a six-fold increase in the odds of atrophy (OR: 6.05; 95% CI: 2.29–15.99; *p* < 0.001). Conversely, smoking was found to be a significant negative predictor, with non-smokers exhibiting a significantly higher risk of advanced mucosal damage (OR: 2.77; 95% CI: 1.22–6.26; *p* = 0.014).

**Figure 2:**
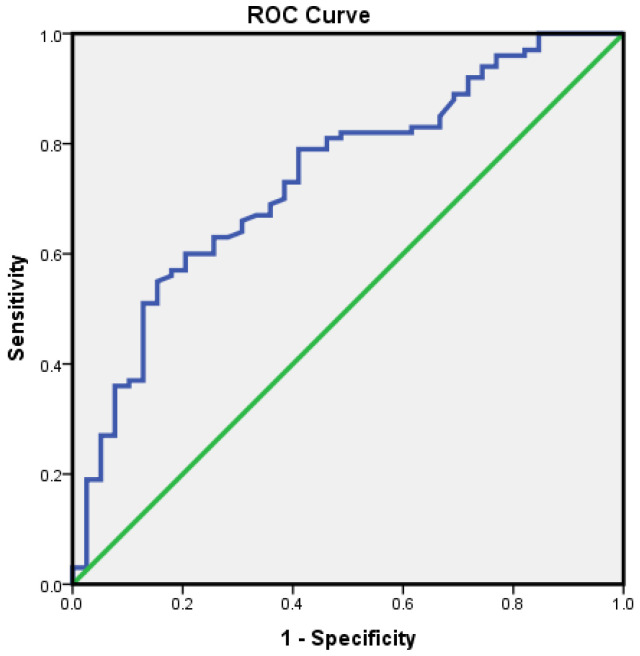
ROC for tTG-IgA in predicting VA (Marsh 3a–c). The AUC is 0.737 (95% CI: 0.646–0.827). Green—Reference line—Chance line; Blue—Diagnostic performance for TTG IGA in predicting VA.

The ROC curve analysis yielded an AUC of 0.737 (95% CI: 0.646–0.827, *p* < 0.001), indicating fair overall discrimination. When applying the prespecified, guideline-driven threshold of ≥10×ULN, the specificity was 84.6% for VA (95% CI: 69.5–94.1%), 90.2% for PPV (95% CI: 78.6–96.7%), and 42.3% for NPV (95% CI: 31.2–54.0%), confirming its utility as a high-confidence “rule-in” tool, despite a moderate sensitivity of 55.0% (95% CI: 44.7–65.0%) (Figure 2).

## 4. Discussion

The translation of the “biopsy-free” approach from pediatric to adult medicine is complicated by the greater clinical and environmental heterogeneity found in adults [13]. While high-titer tTG-IgA is a powerful predictor of mucosal damage, a recognized discrepancy exists between serological magnitude and histological severity in a subset of the adult population [14]. Notably, six patients in our cohort presented with high-titer tTG-IgA, despite demonstrating only mild enteropathy (Marsh 1–2) on histological examination. This discordance between serological and histological findings is not uncommon, and it underscores the inherent limitations of duodenal biopsy as a gold standard. Patchy atrophy, a well-documented phenomenon in adult CD, may result in sampling error where the most severely affected mucosa is bypassed during endoscopy. Furthermore, these cases may represent potential or latent CD, where high serological activity precedes the development of overt VA. Alternatively, a transitioning or healing mucosa in patients who may have inadvertently reduced gluten intake prior to the procedure could account for this discrepancy [15]. These findings suggest that in the presence of extremely high antibody titers, a Marsh 1–2 score should be interpreted with caution and may necessitate a clinical re-evaluation or repeat biopsy to avoid a false-negative histological conclusion.

The main objective of this investigation was to assess the reliability of the non-biopsy method in the adult celiac population. Our results demonstrate that the ≥10×ULN threshold is specific for VA, with a specificity of 84.6% and a PPV of 90.2%. It is essential to interpret our reported predictive values within the context of our clinical setting. As this study was conducted in a tertiary referral center, the pre-test probability and prevalence of CD were significantly higher than in a primary care or general screening population. Because PPV and NPV are inherently dependent on disease prevalence, our high PPV (90.2%) is likely inflated by this high-prevalence environment. Consequently, while the ≥10×ULN threshold remains a robust “rule-in” tool in specialized gastrointestinal units, its PPV may be lower when applied to asymptomatic populations or low-prevalence clinical settings.

Similar results were reported by Penny et al. in a recent paper, which concluded that IgA tTG titers of ≥10×ULN have a strong predictive value at identifying adults with intestinal changes diagnostic of CD [3]. This aligns with several other recent meta-analyses, which suggest that high-titer serology is nearly pathognomonic for CD in adults, just as it is in children [6,9,16]. However, the sensitivity in our cohort was only 55.0%. This indicates that while a high-titer is an excellent “rule-in” tool, it is a poor “rule-out” tool. Nearly half of our patients with confirmed VA would have been missed if clinicians relied solely on reaching the mark. This underscores the continued necessity of the duodenal biopsy for symptomatic patients with lower-range antibody elevations.

The most striking result of our study is the role of smoking as a modifier of histological severity [17]. The “Celiac Paradox”—the observation that smokers are less likely to be diagnosed with CD—is well-documented [18], but our data provides a granular look at how it affects diagnostic discordance. Notably, patients who presented with high antibodies but mild histology (Marsh 1–2) were smokers. This suggests that tobacco use might exert an immunosuppressive effect on the gut mucosa or alter intestinal permeability, effectively masking the development of full VA. Interestingly, our results align with the findings of Wijarnpreecha et al., suggesting that smoking status is a critical modifier that must be integrated into the clinical assessment of suspected CD. Our data support the provocative notion that the biopsy-free approach could offer higher diagnostic accuracy for the smoking subgroup than traditional histology. While high-titer antibodies indicate systemic autoimmunity and active disease, the immunosuppressive or protective effects of tobacco components may mask the development of overt VA on a biopsy [19]. Based on our findings, we agree with the 2023 ACG “conditional recommendation” for a biopsy-free approach in adults, but it could be refined by considering, for example, smoking status. In a non-smoking adult with high tTG-IgA levels, the risk of a false positive is negligible. Conversely, in smokers, clinicians should remain wary of “mild” biopsy results, as they may not reflect the true severity of the autoimmune process.

Our data also highlights the nutritional impact of CD in the Romanian population, with 62.6% of patients suffering from iron deficiency anemia. The association between VA and markers such as hypoalbuminemia and vitamin D deficiency reinforces the idea that adult CD is not merely a digestive issue but a systemic malabsorptive disorder [20]. The prevalence of psychiatric disorders and thyroid disease in our cohort is consistent with the mosaic of autoimmunity often seen in CD. These comorbidities often complicate the clinical picture, making a non-invasive diagnostic pathway even more desirable to reduce the medical burden on these multi-morbid patients.

The present study possesses several key strengths that reinforce the validity and clinical relevance of our findings. First, the sample size of 139 adult patients represents a robust cohort for a single-center study in Romania. While multicenter trials often yield larger numbers, our cohort size provided sufficient statistical power to perform a multivariate logistic regression, allowing us to identify tTG-IgA and smoking status as independent predictors of VA with high confidence. By employing a prospective diagnostic accuracy design rather than a retrospective or case-control approach, we were able to minimize verification bias and more accurately reflect the real-world predictive value of the ≥10×ULN tTG-IgA threshold in an adult population. Second, the homogeneity of our study group is a significant advantage. All patients were managed within a single tertiary referral center, ensuring a consistent diagnostic protocol and clinical management. Furthermore, the robustness of our histological findings is further supported by the standardized evaluation process employed. All histological changes were assessed by two independent, experienced pathologists who were blinded to the clinical and serological data. Discrepancies were resolved through consensus, and grading was strictly performed according to the Marsh–Oberhüber classification. By utilizing two expert observers, we minimized the risk of inter-observer variability, which is a frequent challenge in the interpretation of mild enteropathy (Marsh 1–2) versus true VA. This high level of pathological scrutiny ensures that the identified serology–histology discordance—particularly the 9.2% of patients with high tTG-IgA but preserved villous architecture—represents a reliable finding. It suggests that even under expert microscopic review, a subset of adults with significant systemic autoimmunity does not exhibit the expected mucosal damage, further pointing toward environmental modifiers such as smoking rather than diagnostic error. Ultimately, to the best of our knowledge, this represents the first study to assess the feasibility of a biopsy-free diagnostic approach specifically within an adult Romanian population. By providing localized data, this research fills a critical gap in the Romanian medical literature and offers a validated framework for local clinicians to adopt the 2023 ACG and ESPGHAN-inspired guidelines within our specific healthcare context.

The current study has several limitations that must be taken into account when analyzing the results. While our study adhered to a rigorous diagnostic protocol, the observed mean of 3.4 biopsies per patient falls marginally below the 4–6 samples recommended by international guidelines to definitively exclude patchy VA. It is important to note, however, that a substantial subset of our cohort did receive the optimal 4–6 biopsies as per clinical standards. The overall mean may partially explain why 9.2% of high-titer patients (six out of 61) presented with only mild enteropathy (Marsh 1–2). In these specific cases, the possibility of sampling miss due to the patchy nature of adult CD cannot be entirely discounted. Nevertheless, the ≥10×ULN threshold remains a robust indicator of disease severity in the majority of the adult Romanian population. As a tertiary referral center study, our population may represent a “sickest-of-the-sick” cohort with a higher pre-test probability of CD than what is seen in primary care. This can naturally inflate the PPV, meaning our results might not be perfectly generalizable to asymptomatic screening populations. However, certain limitations regarding tobacco exposure must be acknowledged because this study categorized participants in a binary way—as either smokers or non-smokers—at the time of enrollment, without further quantifying the duration of the habit, the daily intensity (pack-years), or the specific delivery method used, such as traditional cigarettes, vaping, or heat-not-burn products. By grouping current smokers with recent quitters and lacking data on pack-years, there is a potential risk of misclassification that may weaken the inference of smoking as a biological modifier. Smoking intensity and the time elapsed since cessation are known to influence mucosal immunity differently. The lack of detailed smoking topography (different forms of tobacco use result in varying systemic concentrations of nicotine and combustion byproducts—each potentially exerting different degrees of immunosuppressive effects on the intestinal mucosa) remains another limitation of the current study. However, even with this binary approach, the statistical significance of the association remains robust, suggesting a strong underlying biological signal. Future prospective studies should employ more granular categories (never, current, and long-term former smokers) and quantify exposure duration to further elucidate this relationship. Finally, we did not account for the duration of gluten consumption prior to diagnosis. Some patients in the low-titer group might have already initiated a low-gluten or gluten-free diet, which could have suppressed their antibody levels and impacted the correlation analysis.

## 5. Conclusions

In conclusion, our study demonstrates that a tTG-IgA threshold of ≥10×ULN provides a reliable diagnostic surrogate for VA in adult CD. However, it is imperative to emphasize that these findings are specifically applicable to symptomatic Romanian adult cohorts with a high clinical suspicion of CD managed in tertiary care settings. Because diagnostic performance metrics are inherently linked to disease prevalence, the high predictive accuracy observed here (PPV 90.2%) may not be directly generalizable to asymptomatic screening populations or primary care settings where CD prevalence is significantly lower. Consequently, while the threshold serves as a robust “rule-in” tool in high-prevalence clinical contexts, its application in low-prevalence populations requires caution to avoid the risk of false positive diagnoses. Furthermore, the significant role of smoking as a modifier of the sero-histological relationship suggests that a more personalized diagnostic framework—incorporating both serological magnitude and environmental factors—is necessary to bridge the remaining diagnostic gap in the adult population.

## Figures and Tables

**Table 1:** Characteristics of the study cohort.

Patient Characteristic	tTGA < 10×ULN U/mL *n* = 78	tTGA ≥ 10×ULN U/mL *n* = 61	*p*-Value
Gender (F)	62	43	0.221
Residency (U)	57	50	0.217
Mean age (years)	42.3 ± 12.74	40.7 ± 13.26	0.457
CD-associated autoimmune comorbidity	31	23	0.807
First-degree relatives with CD	11	4	0.155
Smoker status (never smoker)	38	19	0.037
**Gastrointestinal Symptoms**			
Abdominal pain	57	47	0.592
Diarrhea	45	43	0.120
Constipation	10	4	0.223
Flatulence	11	26	<0.001
Bloating	36	38	0.058
Dyspepsia	39	33	0.631
Weight loss	55	52	0.041
**Extraintestinal Symptoms**			
Fatigue	45	53	<0.001
Mood changes	13	9	0.759
Anorexia	18	27	0.008
Anemia	42	45	0.016
Aphthous stomatitis	10	12	0.272
Dermatitis herpetiformis	3	4	0.468
Fertility disorders	7	8	0.435
Osteoporosis/osteomalacia	10	7	0.511
**Marsh Classification**			
Marsh 1	9	1	<0.001
Marsh 2	24	0	
Marsh 3a	30	6	
Marsh 3b	11	12	
Marsh 3c	4	41	
**Hematological and Biochemical Parameters**			
Hemoglobin (12–14 g/dL)	11.97 ± 2.13	11.04 ± 2.22	0.013
Platelet count (150,000–450,000/mm^3^)	304,487.17 ± 104,563.12	334,836.06 ± 118,152.89	0.111
Aspartate aminotransferase (8–48 U/L)	36.05 ± 27.58	45.47 ± 26.62	0.042
Alanine aminotransferase (7–55 U/L)	35.43 ± 30.66	49.34 ± 35.68	0.015
Total protein level (6–8.3 g/dL)	7.39 ± 0.75	6.85 ± 0.89	<0.001
Albumin (3.5–5.5 g/dL)	4.07 ± 0.55	3.45 ± 0.62	<0.001
Calcium (8.6–10.4 mg/dL)	8.95 ± 0.69	8.71 ± 0.68	0.037
Iron (15–200 µg/dL)	58.42 ± 45.07	39.88 ± 30.61	0.005
Ferritin (60–170 ng/mL)	96.94 ± 28.37	52.47 ± 47.69	0.270
Vitamin D (30–50 ng/mL)	47.37 ± 41.91	24.49 ± 21.95	0.057
Vitamin B12 (160–95 pg/mL)	314.70 ± 165.56	308.18 ± 169.5	0.822

**Table 2:** Patient characteristics by histological severity (Marsh 3a–c vs. Marsh 1–2).

Characteristic	Marsh 1–2 (*n* = 39)	Marsh 3a–c (*n* = 100)	*p*-Value
Age (years, mean ± SD)	42.12 ± 13.15	41.44 ± 12.82	0.776
Female Gender, *n* (%)	27 (69.23%)	78 (78%)	0.281
Never Smoker, *n* (%)	19 (48.72%)	57 (57%)	0.200
tTG-IgA ≥ 10×ULN, *n* (%)	6 (15.38%)	55 (55%)	<0.001
Hemoglobin (g/dL, mean ± SD)	12.14 ± 1.82	11.41 ± 2.11	0.41
Total IgA (mg/dL, mean ± SD)	214.5 ± 88.2	228.1 ± 92.4	0.422

**Table 3:** Risk factors for VA.

	Univariate Analysis			Multivariate Analysis		
	OR	CI	*p*	aOR	CI	*p*
≥10×ULN tTG-IgA	1.880	1.458–2.426	<0.001	6.055	2.293–15.990	<0.001
Never smoker	1.865	1.283–2.709	0.002	2.774	1.227–6.269	0.014
Age (continuous)	0.992	0.968–1.018	0.760	-	-	-
Gender (female)	1.574	0.695–3.562	0.280	-	-	-

## Data Availability

The data presented in this study are available on request from the corresponding author. The data are not publicly available because they are the property of the Institute of Gastroenterology and Hepatology, Iași, Romania.
